# Luminescence Properties and Mechanisms of CuI Thin Films Fabricated by Vapor Iodization of Copper Films

**DOI:** 10.3390/ma9120990

**Published:** 2016-12-07

**Authors:** Guochen Lin, Fengzhou Zhao, Yuan Zhao, Dengying Zhang, Lixin Yang, Xiaoe Xue, Xiaohui Wang, Chong Qu, Qingshan Li, Lichun Zhang

**Affiliations:** School of Physics and Optoelectronic Engineering, Ludong University, Yantai 264025, China; guochllin@163.com (G.L.); fzzhao@ldu.edu.cn (F.Z.); zhaoyuan062312514@163.com (Y.Z.); dengying_zhang@163.com (D.Z.); lixin_yang0131@163.com (L.Y.); xxe9898@163.com (X.X.); isabellaxiaohui@163.com (X.W.); quchongbjtu@126.com (C.Q.); qsli@ldu.edu.cn (Q.L.)

**Keywords:** CuI thin films, photoluminescence, vapor iodization, iodine vacancy

## Abstract

Copper iodide (CuI) thin films were grown on Si(100) substrates using a copper film iodination reaction method. It was found that γ-CuI films have a uniform and dense microstructure with (111)-orientation. Transmission spectra indicated that CuI thin films have an average transmittance of about 60% in the visible range and the optical band gap is 3.01 eV. By checking the effect of the thickness of the Cu films and annealing condition on the photoluminescence (PL) character of CuI films, the luminescence mechanisms of CuI have been comprehensively analyzed, and the origin of different PL emissions are proposed with Cu vacancy and iodine vacancy as defect levels.

## 1. Introduction

Copper iodide (CuI) is a binary metal halide semiconductor with a cubic zinc-blende structure. Due to the wide direct bandgap of 3.1 eV and a large exciton binding energy (62 meV), CuI has been considered as a promising candidate material for optoelectronic applications, especially for short-wavelength optoelectronic detectors and light emitting devices [1]. Furthermore, CuI has a number of merits, such as p-type conductivity arising from the presence of Cu vacancies, high hole mobility owing to its light hole effective mass, and high transmittance in the visible spectrum. By virtue of these advantageous properties, CuI has been successfully applied in various fields such as transparent conductive films [2], solid-state dye solar cells [3,4], and light-emitting devices [5] for several decades.

Recently, there is a renewed interest in CuI thin films due to their attractive prospects for heterojunction optoelectronic device applications [6,7,8], which has led to a large number of studies of CuI thin films, including growth methods, quality of epitaxial thin films, optical and electrical properties, and device applications [9,10,11,12,13,14,15]. However, in spite of these previous efforts and results, some of the basic properties, such as the defect behavior and the process of carrier recombination in CuI films, still remain unclear. Moreover, the luminescence origin and mechanisms of CuI still needs further investigation.

Different synthesis techniques can be used to prepare CuI thin films [2,11,12,13,14,15,16], such as pulse laser deposition, magnetron sputtering, iodination reaction, vacuum evaporation, and hydrothermal evaporation. Among them, the iodination reaction technique using a copper thin film has attracted enormous attention because of the potential for large area uniform films, low growth temperature, and inexpensive cost [6]. In this paper, we report the fabrication of CuI thin films on Si(100) substrates under different conditions by the copper film iodination reaction technique. Comprehensive photoluminescence (PL) spectral studies, including the effect of the thickness of Cu films and annealing environment, were implemented regarding these different emissions. The defect behavior and luminescence mechanism of CuI thin films are also discussed.

## 2. Experiments

The growth of CuI thin films was carried out in a two-step process. Firstly, the copper thin films were deposited on Si(100) substrates by electron beam evaporation using high purity Cu pellets (99.999%, Kurt J. Lesker Company, Clairton, PA, USA) as the evaporation source. Before depositing the Cu film, the Si(100) wafer was initially cleaned with acetone and ethanol to remove organic grease and then etched with a dilute aqueous HF solution (5.0 mol·L^−1^) for 10 min to remove the native oxide. The growth chamber was evacuated using a turbo molecular pump at 3 × 10^−4^ Pa, and the growth rate of the copper thin films was kept at ~2 Å/s at room temperature. Secondly, the as-fabricated Cu films were transferred to a Petri dish and fixed by a Teflon holder. Iodine particles (99.999%, Alfa Aesar, Ward Hill, MA, USA) were put into the Petri dish and then heated to 150 °C for 1 h by a hotplate.

The thickness of the Cu thin films was determined by a Dektak XT profilometer (Bruker, Karlsruhe, Germany). A NanoNova 450 scanning electronic microscope (SEM, FEI, Hillsboro, OR, USA) was used to examine the morphology of the samples. The X-ray diffraction (XRD) measurements were performed on Rigaku D/MAX2500V diffractometer (Rigaku, Osaka, Japan) with Cu Kα radiation (λ = 0.15405 nm). Photoluminescence (PL) spectra were excited by a 325 nm He-Cd laser (Kimmon Electric, Ltd., Saitama, Japan) at room temperature. A monochromator (SR-500i-A, Andor Technology, Belfast, UK) was employed for collecting the PL emission spectra. All the results were measured at room temperature.

## 3. Results and Discussion

**Figure 1:**
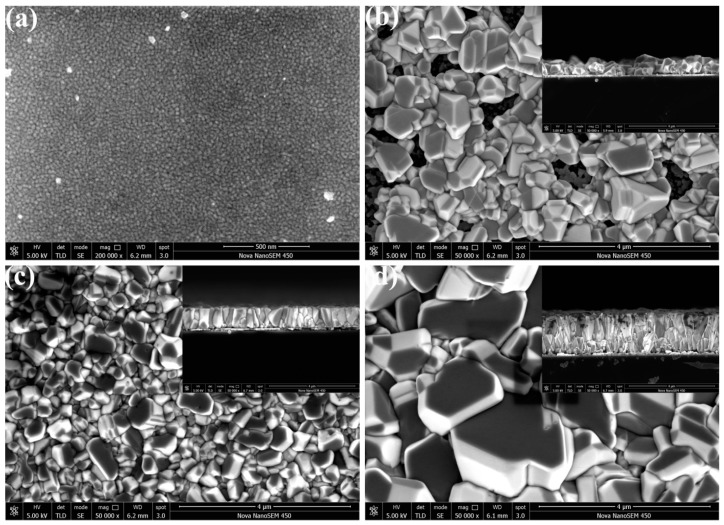
(**a**) Surface SEM images of the Cu films. Surface and cross-sectional (inset) view SEM images of the CuI thin films grown with different thickness of Cu films (**b**) 80 nm; (**c**) 200 nm; (**d**) 500 nm.

Figure 1a shows the surface SEM images of the Cu thin films grown on Si(100) substrates. The thickness of the Cu thin films was determined by a Bruker Dektak XT profilometer to be about 80 nm. It can be observed that the Cu films grown by electron beam evaporation possess the crystalline size of nanometer order. The surface SEM images in Figure 1b–d illustrate CuI thin films grown under different thickness of Cu films (80 nm, 200 nm, 500 nm, respectively). The corresponding cross-sectional SEM images are shown in the insets of Figure 1b–d, respectively. Compared with the surface SEM images of the Cu films (Figure 1a) and CuI films (Figure 1b), evident change are easily observed. All of the CuI films exhibit a very rough surface morphology, consisting of many small crystallites with facets in different growth orientations [6]. As shown in the cross-sectional SEM images, microcolumnar structures are vertically arranged on the Si(100) substrate. In comparison with the Cu thin film, the thickness of all of the CuI films increased significantly (nearly five times), resulting from the theoretical volume ratio of CuI and Cu (4.76) [6]. A typical atomic force microscopy (AFM) scan is depicted in Figures S1 and S2, revealing a very rough surface morphology for the CuI thin films grown using 200 nm thickness of the Cu films (see Supplementary Materials).

**Figure 2:**
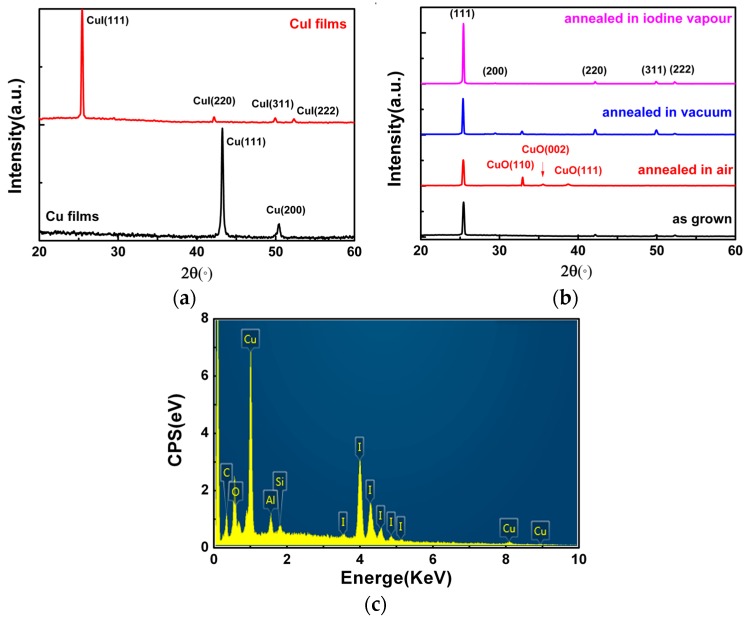
(**a**) XRD patterns of Cu films and CuI films; (**b**) XRD patterns of CuI films with and without annealing treatment; (**c**) EDXA result of the CuI films.

As the CuI grown under 200 nm Cu films had a uniform surface texture, it was used for the growth of CuI for XRD measurements. Figure 2a shows XRD patterns of Cu and CuI thin films, in which the thickness of the Cu films was 200 nm. The XRD result of the as-deposited Cu film grown on Si(100) substrate indicates that there were two diffraction peaks at 43.24° and 50.44°, corresponding to Cu(111) and Cu(200), respectively. The XRD pattern of the as-grown CuI films exhibits peaks corresponding to the (111), (220), (311), and (222) planes of CuI in zinc-blende structure (JCPDS card No. 060246) [2,10,17]. Figure 2b shows XRD patterns of CuI thin films with diferent annealing treatments. The CuI films were grown under the same condition (the thickness of the Cu films was 200 nm) and the annealing treatment was performed in a quartz tube under different conditions (air, vacuum, and iodine vapors) at 300 °C for 2 h. For CuI thin films annealed in air, vacuum, and iodine vapors [10], the intensity and full width at half maximum (FWHM) of the CuI(111) were changed. Under different environments, the FWHM of the CuI(111) decreases from 0.213° (as grown) to 0.207° (air), 0.163° (vacuum), and 0.157° (iodine vapors), respectively. It is indicated that the crystallization quality of CuI thin films has been improved through the annealing treatment [18]. However, in addition to the CuI(111), three additional peaks are also found in the XRD spectrum of CuI films annealed in air, which correspond to CuO(110), (002), and (111) (JCPDS card No 45-0937) [19]. This can be attributed to the fact that an oxidation reaction occurred between the CuI films and oxygen.

Quantitative analysis of the chemical compositions of the CuI thin films was further performed by surface scanning EDXA (Energy Dispersive X-ray Analysis). As shown in Figure 2b, the elemental ratio (at %) of the Cu/I is 1.06:1, which is near the standard stoichiometric composition [17]. Furthermore, other elements have also been found in the CuI thin films, including Al, Si, C, and O. It was deduced that Si comes from the Si(100) substrate and the Al signal observed in the spectrum arises from Al contamination in the vacuum chamber. The appearance of O and C might be attributed to H_2_O, O_2_, and CO_2_ adsorbed on the surface of the CuI crystals in air [17]. Though the EDXA for this kind of quantitative analysis is shown, X-ray photoemission spectroscopy (XPS) will be further explored in subsequent works.

**Figure 3:**
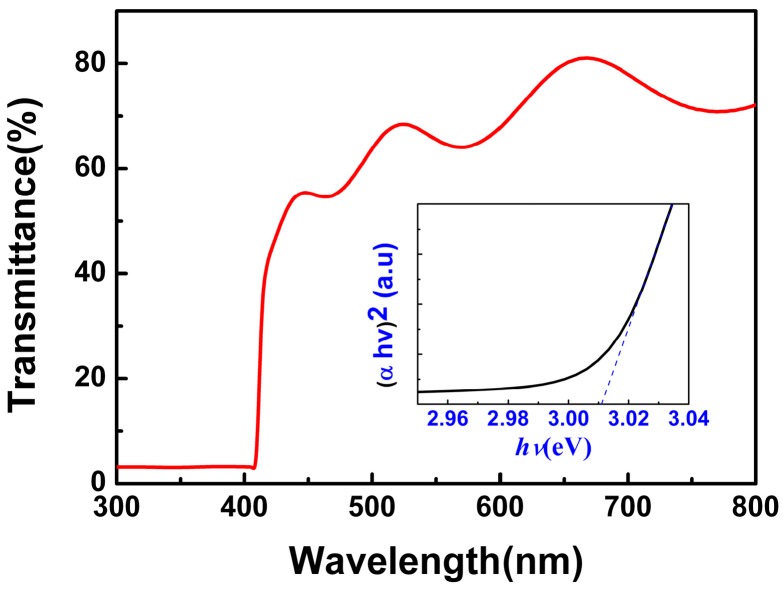
Transmission spectra of the CuI thin films, the inset shows the optical band gap of CuI thin films grown on glass substrate.

Figure 3 shows the transmission spectra of the CuI thin films grown under 80 nm thickness Cu films (glass substrate). The optical transmittance of CuI thin films is about 60% in the visible wavelength range (400–800 nm). The optical band gap (*E*_g_) was calculated by using the relationship $\alpha^{2}=A\left(hv-E_{g}\right)$, where α and *hν* are the absorption coefficient and the photon energy, respectively [10], and A is a constant. The values of the theoretical band gap were determined by the extrapolation of linear regions of the plots to zero absorption (α*hv* = 0). The optical band gap of the as-grown CuI thin films, obtained from the inset of Figure 3, was 3.01 eV, which is consistent with the results in previous works [2,10].

**Figure 4:**
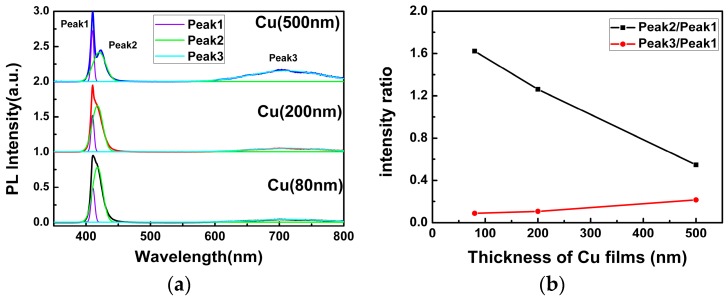
(**a**) Photoluminescence (PL) spectra of the as-grown CuI samples under different thickness of Cu films; (**b**) The intensity ratios of the different PL peaks.

Figure 4a shows the PL spectra of the as-grown CuI samples, which have been iodinated by different thicknesses of Cu films under the same conditions. All of them exhibit a strong blue emission band and a weak broad red emission band. In order to better understand the origin of the PL emission, the Gaussian fits of the PL spectra are shown in Figure 4a. It can be seen that the blue emission band can be divided into two luminescent peaks, which are centered at ~410 nm (Peak 1) and ~419 nm (Peak 2), respectively. This phenomenon can be observed directly in the PL spectra of CuI grown using 500 nm Cu films and other previous reports [9,10,18]. Generally speaking, the emission of Peak 1 with 3.02 eV is reasonably ascribed to radiative recombination of free excitons [10,13]. However, there is still much debate on the origin of the emission of Peak 2 with 2.96 eV and the broad red emission band around 715 nm (Peak 3). One theory claimed that the emission of Peak 2 with 2.96 eV is attributed to the excessive iodine accumulated on the CuI surface [20,21], while others have postulated that it originated from the bulk iodide vacancies [9]. Meanwhile, the intrinsic Cu vacancies have also been speculated as the potential sources for the near-band emission (Peak 2) [10,22]. For the origin of the red emission (Peak 3), some have argued that it is induced by the oxygen vacancies [15], while the majority still consider it to be related to the iodine vacancy [10,23].

In order to investigate the origin of the Peak 2 and Peak 3 emissions in CuI films, Figure 4b shows the intensity ratio of Peak 2 and Peak 3 relative to Peak 1. The intensity ratio of Peak 2 to Peak 1 decreased substantially with the increasing thickness of Cu films, whereas the intensity ratio of Peak 3 to Peak 1 increased slightly. It is important to note that the iodination reaction occurred in iodine-enriched environments, and the Cu vacancies appeared frequently [10], acting as an acceptor level in CuI. As a result, the electron transfer from the conduction band minimum (CBM) to the Cu vacancy acceptor level and the emission band centered at ~419 nm (Peak 2) was observed. As can be seen from Figure 4b, since the concentration of Cu vacancy decreased with the increasing thickness of Cu films, the intensity of Peak 2 reduced. However, the change of the intensity ratio of Peak 3 to Peak 1 is too slight to confirm whether the origin of the red emission is related to the iodine vacancy or the oxygen vacancy.

**Figure 5:**
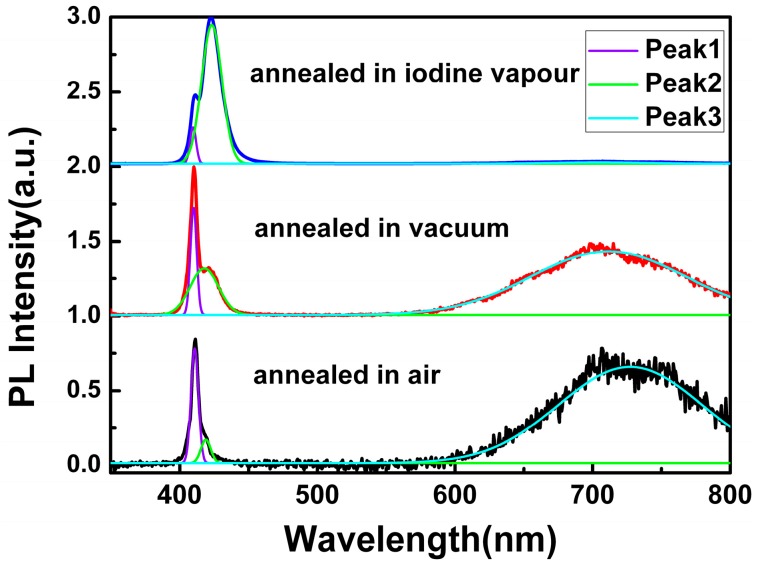
PL spectra of CuI thin films annealed in different environments.

To get a comprehensive understanding of the origin of the red emission, annealing treatment for CuI films grown under the same conditions (the thickness of Cu films was 200 nm) was performed under air, vacuum, and iodine vapor conditions, respectively. Figure 5 displays the PL spectra of the CuI thin films annealed in different environments, while the peak-deconvolution with Gaussian function was applied to each spectrum. In comparison, the PL parameters of the CuI thin films with and without annealing treatment, such as the FWHM of Peak 1 and the intensity ratio of different PL peaks, are shown in Table 1.

As can be seen in Figure 5 and Table 1, the FWHM of Peak 1 decreased after annealing treatment in vacuum and iodine vapor, which indicated that the annealing environment of CuI thin films in vacuum and iodine vapor can effectively reduce film defects [10,18,24]. However, for the CuI thin films annealed in air, the FWHM of Peak 1 increased in comparison to the as-grown CuI thin films without annealing treatment, which is probably due to the oxidation reaction occurring in the annealing treatment under air.

According to the changes of the intensity ratio of Peak 2 to Peak 1, the value decreased when the CuI thin films were annealed in air and vacuum, but was dramatically enhanced by iodine annealing. As the mobility of iodine ions in the crystal lattice of CuI becomes more active at high annealing temperatures, iodine can escape from the material, leading to a reduction of Cu vacancy [10]. As a result, the intensity of Peak 2 related to the Cu vacancy acceptor level decreased when annealing in air and vacuum. Conversely, iodine annealing can increase the amount of Cu vacancies, and the intensity of the Peak 2 improved.

Finally, the origin of the red emission can be deduced by examining the changes of *I*_peak3_/*I*_peak1_. Compared with the as-grown CuI thin films, the intensity of Peak 3 obviously enhanced when annealed in air, which implies that the red emission should not be related to the oxygen vacancy. However, since iodine can escape from CuI, and Cu should be oxidized when annealed in air, it can be inferred that the red emission could be induced by the iodine vacancy. Furthermore, the value of *I*_peak3_/*I*_peak1_ also improved by vacuum annealing without oxygen participation, which is further proof of the relationship between the iodine vacancy and the red emission [10,23]. Additionally, the red emission almost disappeared after iodine annealing, indicating that the lack of iodine in the as-grown CuI thin films can be supplemented through annealing the films in iodine vapors [10]. In conclusion, it can be confirmed that the red emission in CuI films can be attributed to the iodine vacancy.

**Figure 6:**
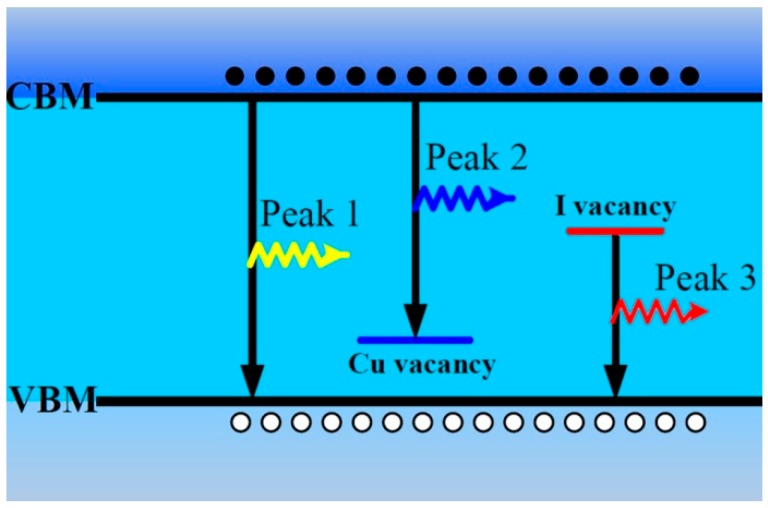
Proposed mechanisms of PL in CuI thin films.

According to the abovementioned results about the PL properties of CuI thin films [25], we propose their origin as shown in Figure 6. The emission of Peak 1 near the band edge with 3.02 eV is reasonably ascribed to free exciton recombination [10,13]. The emission of Peak 2 with 2.96 eV is ascribed to the electron transition from the CBM to Cu vacancy acceptor level, and the intensity of Peak 2 can be improved by iodine annealing [10]. According to the experimental results in this paper and other previous reports [10,23], the red emission band can be attributed to the transition from iodine vacancy deep donor level to the value band maximum (VBM). These defects and PL bands can be formed during the air or vacuum annealing process.

## 4. Conclusions

In summary, CuI thin films were deposited onto Si(100) substrates via iodination reaction with Cu films. The morphology, structure, composition, and luminescence characteristics of the CuI thin films have been investigated by SEM, XRD, EDXA, and PL. It is shown that CuI thin films have a (111) preferred orientation, and a 60% average transmittance in the visible range can be obtained. A comprehensive interpretation for the origin of the different PL emission bands has been obtained by analyzing the effect of thickness of the Cu films and the annealing environment. It is believed that the 419 nm emission is ascribed to the electron transition from the CBM to the Cu vacancy acceptor level, whereas the red emission band is related to iodine vacancies.

## Figures and Tables

**Table 1:** PL parameters of CuI thin films with and without annealing treatment.

Sample (Annealing Treatment)	FWHM of Peak 1	*I*_peak2_/*I*_peak1_	*I*_peak3_/*I*_peak1_
as-grown	5.586	1.262	0.106
Air	6.361	0.219	0.859
vacuum	5.366	0.438	0.598
iodine vapor	5.243	3.582	0.008

## Supplementary Materials

The following are available online at www.mdpi.com/1996-1944/9/12/990/s1. Figure S1: AFM image of the CuI thin film, Figure S2: Roughness analysis for the AFM image of the CuI thin film.
