# Supplementary material for: Luminescence Properties and Mechanisms of CuI Thin Films Fabricated by Vapor Iodization of Copper Films
Source: Materials (Basel). 2016 Dec 7;9(12):990. doi: 10.3390/ma9120990 (PMC5456966; doi:10.3390/ma9120990)
Supplement: Supplementary file 1 [file materials-09-00990-s001.pdf]

Supplementary Materials: Luminescence Properties and Mechanisms of CuI Thin Films Fabricated by Vapor Iodization of Copper Films

Guochen Lin, Fengzhou Zhao, Yuan Zhao, Dengying Zhang, Lixin Yang, Xiaoe Xue, Xiaohui Wang, Chong Qu, Qingshan Li and Lichun Zhang

A typical atomic force microscopy (AFM) scan is depicted in Figure S1, revealing a very rough surface morphology for the CuI thin films grown under 200 nm thickness of Cu films.

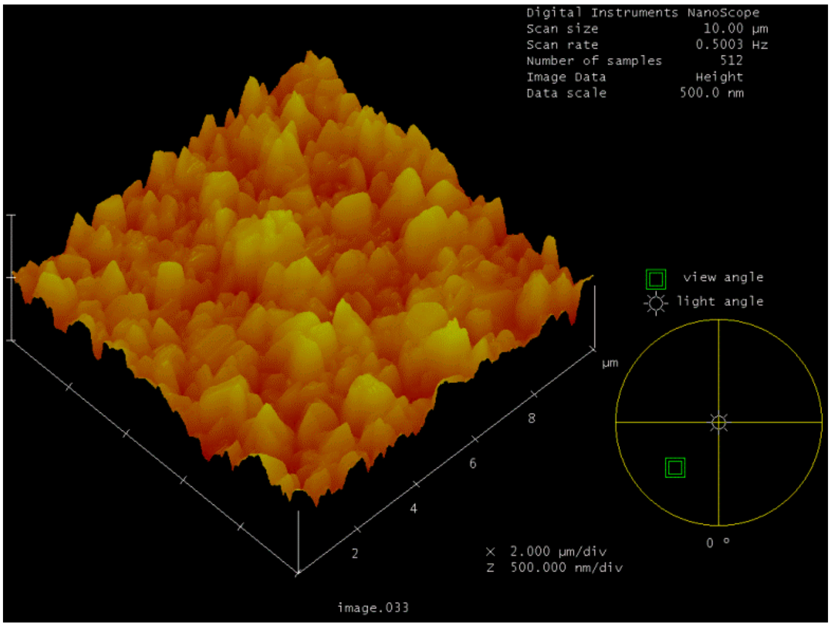

Figure S1. AFM image of the CuI thin film.

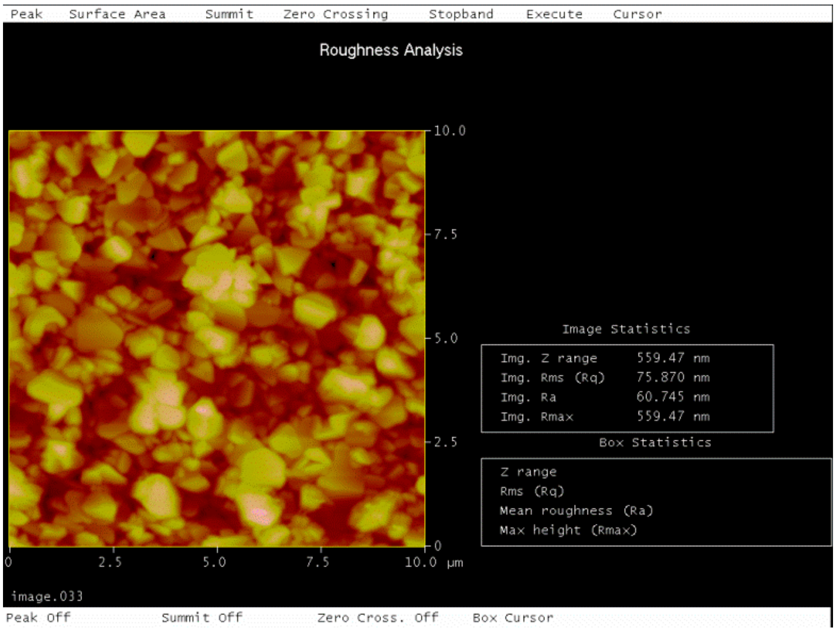

Figure S2. Roughness analysis for the AFM image of the CuI thin film.
